# Probable HTLV-I/II Tropical Spastic Paraparesis Patient from Ethiopia: A Case Report

**DOI:** 10.4314/ejhs.v32i4.24

**Published:** 2022-07

**Authors:** Dereje Melka, Mehila Zebenigus

**Affiliations:** 1 Department of Neurology, Addis Ababa University School of Medicine, Addis Ababa, Ethiopia; 2 Yehuleshet Neurology specialty Clinic, Addis Ababa, Ethiopia

**Keywords:** Probable HTLV associated myelopathy/ Tropical Spastic Paraparesis, case report, Ethiopia

## Abstract

**Background:**

Available data on the burden of Human T-cell lymphotropic virus type I/II infection for eastern Africa, limited to Ethiopia, Mozambique, and Rwanda, show prevalence lower than elsewhere in Africa (0% – 1.8%). Even if Tropical Spastic Paraparesis occurs in an endemic form in Ethiopia, its seroprevalence is low. Over a lifetime, it is estimated that 1–2% of Human T-cell lymphotropic virus type I/ II -infected individuals will develop progressive and disabling inflammatory clinical manifestations. We are reporting this case since it signifies the existence of seropositive Tropical Spastic Paraparesis in our setting and the need to properly diagnose this condition.

**Case Presentation:**

We are reporting a 45 years old female patient from Addis Ababa, Ethiopia, who presented with progressive weakness of the lower limbs and urinary urge incontinence of five years duration. Serology for Human T-cell lymphotropic virus type I/ II antibody was positive. She was diagnosed to have probable tropical spastic paraparesis after fulfilling World Health Organization diagnostic criteria for tropical spastic paraparesis with the level of ascertainment. Symptoms showed transient improvements after providing five days of Methylprednisolone followed by low doses of corticosteroids and Azathioprine. The patient is now significantly disabled and wheelchair-bound.

**Conclusions:**

The patient described here signifies a probable Human T-cell lymphotropic virus type I/ II - associated myelopathy/tropical spastic paraparesis in Ethiopian women. This case highlights the existence of Human T-cell lymphotropic virus type I/II - associated myelopathy/ tropical spastic paraparesis within our setting and the need to properly diagnose this condition.

## Introduction

Human T-cell lymphotropic virus type I/II (HTLV-I/II)-associated myelopathy/tropical spastic paraparesis (HAM/TSP) is a rare chronic neuroinflammatory disease that develops in a small proportion of those infected with HTLV-I/II (1). HTLV-I/II infection is initially asymptomatic and can last for several years or even decades.

Few patients subsequently develop inflammatory and malignant diseases that commonly present with weakness or paralysis of the legs, lower back pain, and urinary symptoms (1).

Available data on the burden of HTLV-1 infection for eastern Africa, limited to Ethiopia, Mozambique, and Rwanda, show prevalence lower than elsewhere in Africa( 0% – 1.8%) (2). Even if TSP occurs in an endemic form in Ethiopia, its seroprevalence is low (3). In our setting, there are major difficulties in evaluating patients with TSP/HAM because of the lack of neurologists, laboratories, and radiologic explorations. Our case fulfills levels of ascertainment for diagnostic criteria of tropical spastic paraparesis/HTLV-1-associated myelopathy (TSP/HAM) Table 1(4).

**Table 1:** Levels of ascertainment for diagnostic criteria of tropical spastic paraparesis/HTLV-1-associated myelopathy (TSP/HAM)

**Definite**

1. A non-remitting progressive spastic paraparesis with a sufficiently impaired gait to be perceived by the patient. Sensory symptoms or signs may or may not be present. When present, they remain subtle and without a clear-cut sensory level. Urinary and anal sphincter signs or symptoms may or may not be present. 2. Presence of HTLV-I antibodies in serum and CSF confirmed by Western blot and/or a positive PCR for HTLV-I in blood and/or CSF 3. Exclusion of other disorders that can resemble TSP/HAM.

**Probable** 1. Monosymptomatic presentation: spasticity or hyperreflexia in the lower limbs or isolated Babinski sign with or without subtle sensory signs or symptoms, or neurogenic bladder only confirmed by urodynamic tests. 2. Presence of HTLV-I antibodies in serum and/or CSF confirmed by Western blot and/or a positive PCR for HTLV-I in blood and/or CSF. 3. Exclusion of other disorders that can resemble TSP/HAM.

**Possible** 1. Complete or incomplete clinical presentation. 2. Presence of HTLV-I antibodies in serum and/or CSF confirmed by Western blot and/or a positive PCR for HTLV-I in blood and/or CSF. 3. Disorders that can resemble TSP/HAM have not been excluded.

To our knowledge, there have been very limited recent cases of seropositive HAM/TSP published in sub-Saharan Africa and there was no recent seropositive case report from Ethiopia. We are reporting this case since it signifies the existence of seropositive HAM/TSM in our setting and the need to properly diagnose this condition plus to consider it as a differential diagnosis of non-compressive myelopathy.

## Case Presentation

**History**: The patient was a 45 years old female from Addis Ababa, Ethiopia. She presented to one of the private clinics at Addis Ababa on May 6, 2017, with progressively worsening weakness of the lower extremities for one year before the current presentation. She also had associated urinary urge incontinence of the same duration. Otherwise, there was no personal or family history of diabetes or cardiac illness. No history of cough, night sweat, and weight loss was recorded. She had no history of exposure to toxins and travel history out of Ethiopia.

**Physical examination**: The general systemic examinations were normal. Blood pressure ranged from 110/70 to 170/90 mmHG other vital signs were within the normal range. Mental status exam: alert and oriented with fluent language and intact comprehension. Cranial nerves: there were no cranial nerve abnormalities detected. Motor: muscle bulk and tone were normal; however, power on lower limbs were 2/5 to 3/5 bilaterally which was prominent on the distal part; reflex +¾ both on ankle and knee, and plantar responses were up going bilaterally. The upper limb motor examination was normal. Sensory: sensation was intact for light touch, pinprick, vibration, and position throughout. Coordination: No coordination abnormalities were detected in the upper limbs, however difficult to test coordination in the lower limbs because of the weakness. Meningeal irritation signs were also negative.

**Auxiliary examinations**: Her complete blood count, erythrocyte sedimentation rate, liver function test, lipid profile, renal function test, serum electrolytes, CSF analysis, serum copper level, Serum vitamin B12 level, Serum folate level, and plasma glucose levels were normal. Serum VDRL was non-reactive; HBsAg and anti HCV antibodies were negative and serology for retroviral infections was also negative. Serum Antinuclear antibody (ANA) was also negative. The serum antibody test for HTLV I/II was positive (Enzyme-linked assay and Western blot) done at Bioscientia Institute for Medical Diagnostics GmbH in Germany.

**Figure 1:**
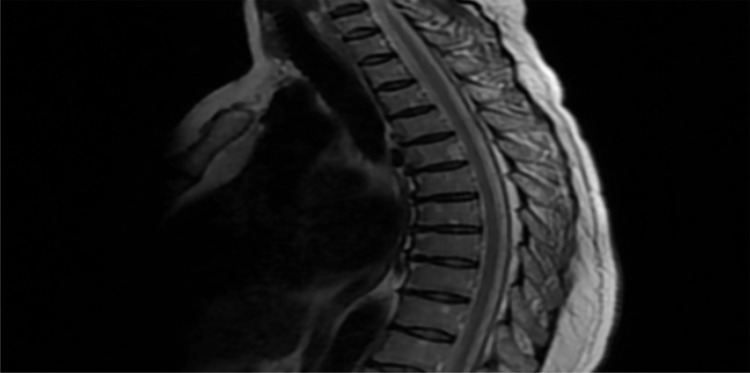
Magnetic resonance imaging studies. Axial T2-weighted Magnetic resonance imaging of the thoracic cord showed normal finding.

NCS and EMG study revealed chronic neurogenic changes in the bilateral L5/S1 myotome with few acute ongoing denervation changes on the left L5 myotome. Her brain and cervical MRI results were normal. Thoracic MRI was also normal (Figure 1). Lumbosacral MRI revealed L4/L5 mild disc dehydration and mild central disc bulge.

**The course of treatment**: She was treated with methylprednisolone 1mg Intravenous (IV) daily for 5 days plus physical therapy after which she had significant improvement of the lower limb weakness. However, after a year of the first visit, she presented with worsening weakness of the lower limbs plus burning pain for 2 months duration. She had become wheelchair-bound and could only walk with bilateral support. She was started on Azathioprine 100mg PO daily and prednisolone 10mg PO BID, which gradually tapered off. She had also developed hypertension for which she was on Lisinopril 10mg PO daily. Nine months after the second visit, she was able to walk with a walking stick. She was on Azathioprine 100mg PO daily and doing regular exercise. Over the next two years, however, the lower extremity weakness, bowel and bladder dysfunction worsened, and became wheelchair-bound.

## Discussion

Even if, TSP occurs in an endemic form in Ethiopia, its seroprevalence is low(3). To our knowledge, there have been very limited recent cases of seropositive HAM/TSP published in sub-Saharan Africa and there was no recent seropositive case report from Ethiopia. This case shows the existence of HTLV I/II seropositive TSP occurring in Ethiopian patients. The previously reported HTLV I/II seropositive HAM/TSP case in Ethiopia (3) was thirty-one years back. This makes our case the most recent case of HTLV I/II seropositive HAM/TSP patient from Ethiopia.

Patients with HTLV I/II seropositive HAM/TSP develop a significant disability and a serious quality of life decline due to gait disturbance, urinary dysfunction, as well as numbness, and pain in their lower limbs (1). This is evidenced in our patient that she developed spastic paraparesis and urge incontinence.

Therefore, a high index of suspicion and considering it as a differential diagnosis of non-compressive myelopathy are essential to make this diagnosis early and demonstrate the need for timely supportive care. Neurosyphilis, subacute combined spinal cord degeneration (vitamin B12 deficiency), and vacuolar myelopathy are some of the mimickers of the clinical presentation of HAM/TSP. Even if the clinical presentation of HAM/TSP is similar to the above differential diagnosis, the absence of laboratory evidence made those differential diagnoses unlikely.

The patient described here signifies a probable case of HTLV I/II seropositive HAM/TSP occurring in this Ethiopian woman. The low expectation of such diseases in our setting, together with diagnostic difficulties, often leads to misdiagnosis.

No therapy has been conclusively shown to modify the long-term disability associated with HAM/ TSP. Clinical improvements have been reported for several agents in open-label studies (corticosteroids, plasmapheresis, danazol, pentoxifylline, and interferon). Except for the interferon-α, the other drugs still lack the quality of evidence required to merit a strong recommendation for their use in HAM/TSP. However, the long-term benefit of interferon-α remains in question, as there are no conclusive studies (5). Since interferon-α is not available in our setup, our patient was given only intravenous methylprednisolone, oral Prednisolone, and Azathioprine during her treatment course.

The disease usually progresses without remission, but there is individual variation in the rate of disease progression. Early diagnosis and prompt treatment are essential for better management of patients with the disease. In a 14-year clinical follow-up study in Martinique, the median time from onset to use of a unilateral walking aid was 6 years; the median onset to wheelchair dependency was 21 years (1). However, our patient became wheelchair-bound after 4 years of symptom onset. This could probably be due to a delay in diagnosis.

Finally, this case highlights the existence of HTLV I/II seropositive HAM/TSP within our setting and the need to properly diagnose this condition even in a resource-limited setting. We believe that the awareness offered here may be valuable to several other resource-limited situations. We recommend routine HTLV I/II serologic screening of patients with typical clinical presentations of HAM/TSP.
